# Detecting Potential Adverse Drug Reactions Using a Deep Neural Network Model

**DOI:** 10.2196/11016

**Published:** 2019-02-06

**Authors:** Chi-Shiang Wang, Pei-Ju Lin, Ching-Lan Cheng, Shu-Hua Tai, Yea-Huei Kao Yang, Jung-Hsien Chiang

**Affiliations:** 1 Department of Computer Science and Information Engineering National Cheng Kung University Tainan Taiwan; 2 School of Pharmacy College of Medicine National Cheng Kung University Tainan Taiwan; 3 Institute of Clinical Pharmacy and Pharmaceutical Sciences College of Medicine National Cheng Kung University Tainan Taiwan; 4 Department of Pharmacy National Cheng Kung University Hospital National Cheng Kung University Tainan Taiwan; 5 Institute of Medical Informatics National Cheng Kung University Tainan Taiwan

**Keywords:** adverse drug reactions, deep neural network, drug representation, machine learning, pharmacovigilance

## Abstract

**Background:**

Adverse drug reactions (ADRs) are common and are the underlying cause of over a million serious injuries and deaths each year. The most familiar method to detect ADRs is relying on spontaneous reports. Unfortunately, the low reporting rate of spontaneous reports is a serious limitation of pharmacovigilance.

**Objective:**

The objective of this study was to identify a method to detect potential ADRs of drugs automatically using a deep neural network (DNN).

**Methods:**

We designed a DNN model that utilizes the chemical, biological, and biomedical information of drugs to detect ADRs. This model aimed to fulfill two main purposes: identifying the potential ADRs of drugs and predicting the possible ADRs of a new drug. For improving the detection performance, we distributed representations of the target drugs in a vector space to capture the drug relationships using the word-embedding approach to process substantial biomedical literature. Moreover, we built a mapping function to address new drugs that do not appear in the dataset.

**Results:**

Using the drug information and the ADRs reported up to 2009, we predicted the ADRs of drugs recorded up to 2012. There were 746 drugs and 232 new drugs, which were only recorded in 2012 with 1325 ADRs. The experimental results showed that the overall performance of our model with mean average precision at top-10 achieved is 0.523 and the rea under the receiver operating characteristic curve (AUC) score achieved is 0.844 for ADR prediction on the dataset.

**Conclusions:**

Our model is effective in identifying the potential ADRs of a drug and the possible ADRs of a new drug. Most importantly, it can detect potential ADRs irrespective of whether they have been reported in the past.

## Introduction

An adverse drug reaction (ADR) [1,2] is a serious problem that refers to side effects that occur despite the administration of a regular dose of a drug. It is estimated that over 2 million serious ADRs occur among hospitalized patients, which causes >100,000 deaths each year [3,4]. Unfortunately, it is difficult to identify or predict potential ADRs owing to insufficient data.

Spontaneous reporting in pre- and postmarket stages are the most familiar methods to identify ADRs early on. Specifically, safety reports from clinical trials are used to list common ADRs in the premarket stage [5], while data collected and analyzed from various databases and marketing surveys, such as pharmacovigilance and risk management, are used in the postmarket stage. Although most new ADRs are identified from spontaneous reports, >90% go unreported [6,7]; this is recognized to be a major limitation. Pharmaceutical companies are trying to avoid side effects in the development stage of drugs. However, although they can identify and address common side effects, it is generally not feasible to identify or predict rare and serious side effects. To overcome these limitations, several studies have utilized a substantial amount of data and various information sources to predict ADRs using statistical methods [8] and machine learning approaches [9].

The fundamental method for identifying ADRs pertains to identifying the relationship between drugs and their side effects from diverse sources of information [10-12] such as clinical trials, electronic medical records (EMRs), social media, and biomedical literature. For instance, PubMed contains valuable information that could aid ADR detection. Karimi et al [13] reviewed data mining and techniques related to computer science, which have been studied in the area of drug safety to identify reports of ADR from different sources. Tatonetti et al [14] proposed a novel algorithm for building a predictive model that can detect hidden interactions in adverse event reports to infer unreported adverse events. Wang et al [15] developed a model for identifying ADRs using data mining to extract information from millions of EMRs. It used clinical notes with information on specific drugs and known adverse drug events (ADEs) that have been preprocessed using statistical methods to compute the probability that a given drug-disorder pair represents a valid ADE association. This method automatically determines whether a specific adverse event is caused by a specific drug based on the content of PubMed citations [16]. Finkelstein et al [17] developed a tool to automatically detect and summarize information on ADRs from journal papers. It then ranked the ADRs of a drug on a user-friendly interface for physicians.

Several studies have utilized either chemical or molecular pathways of drugs to predict ADRs [18]. Cami et al [19] developed a novel approach to predict ADEs by using information on specific drugs and the adverse event to predict likely unknown ADEs. Lorberbaum et al [20] hypothesized that systems biology and chemical genomics data can improve drug safety surveillance by highlighting drugs with a mechanistic connection to the target phenotype and by filtering those which do not. They presented an algorithm, the modular assembly of drug safety subnetworks, to combine systems pharmacology and pharmacovigilance data. The algorithm markedly improved drug safety monitoring for 4 clinically relevant ADRs. Huang et al [21] proposed a framework for predicting ADR profiles by integrating protein-protein interaction networks with drug structures. Some researchers utilized the chemical, biological, and phenotypic characteristics of drugs to predict the ADRs. Liu et al [22] proposed a machine learning approach for predicting the ADRs by integrating the phenotypic characteristics, which included chemical structure, biological properties, and protein target and pathway information.

However, most of those approaches rely on heavily handcrafted features and treat ADR identification as a classification problem, which does not take the order of the ADRs discovered into consideration. Therefore, the process tends to be more expensive and leads to the loss of significant information on drug-ADR relationships in the model training phase. Furthermore, these approaches are unable to predict the ADR of new drugs, thus rendering the detection of ADR more difficult [19].

To address these limitations, we used a deep neural network (DNN) model for the detection of ADRs of drugs. The model has 2 purposes: the identification of ADRs, which entailed the discovery of potential ADRs of a drug from known ADR records, and the prediction of ADRs, which pertained to predicting the possible ADRs for a new drug. We used the word-embedding approach and mapping function to process new drugs that did not appear in the dataset. Furthermore, we examined the overall performance of the model with various feature combinations and the number of hidden layers in the DNN architecture.

## Methods

### Data Description

To develop and evaluate a DNN model, we used data from Side Effect Resource (SIDER) [23], a database of drugs with side effects, which contains information on medicines in the market since 2009 and their recorded ADRs [24]. We collected the ADR information from 2009 and 2012 from SIDER to represent the simulated prospective approach. In total, 746 drugs and 1325 side effect terms related to these drugs were recorded in both years. Additional 232 drugs appeared only in the 2012 dataset as new drugs. It is important to monitor the ADRs of drugs throughout their life cycle, from the preclinical research phase to postmarket surveillance. The fundamental properties of drugs rely on preclinical *in vitro* safety profiling that involves the testing of compounds with chemical and biological properties. Therefore, we extracted these properties as a part of the features in the model. We extracted the 17 molecular descriptors of drugs from PubChem [25] (Textbox 1). We utilized the biological features from DrugBank [26] to represent the biomolecular interactions and pathways. These features contain the targets, enzymes, transporters, and carriers of each drug and their actions.

For enriching the scientific evidence and enhancing the detection of ADRs, we collected millions of papers from the Medical Literature Analysis and Retrieval System Online (MEDLINE) [27] to be used as auxiliary data to enrich the information about each drug. We used the name of each drug as the query term and selected all the papers related to the drug, published before 2009, such as case reports, clinical trials, and observational studies. The reason for collecting the papers published before 2009 is that we wanted to simulate the progress of drug surveillance from 2009 to 2012.

**The 17 molecular descriptors for the chemical features identified in this study.**

1. Molecular Weight
2. XLogP3
3. Hydrogen Bond Donor Count
4. Hydrogen Bond Acceptor Count
5. Rotatable Bond Count
6. Exact Mass
7. Monoisotopic Mass
8. Topological Polar Surface Area
9. Heavy Atom Count
10. Formal Charge
11. Complexity
12. Isotope Atom Count
13. Defined Atom Stereocenter Count
14. Undefined Atom Stereocenter Count
15. Defined Bond Stereocenter Count
16. Undefined Bond Stereocenter Count
17. Covalently- Bonded Unit Count

### Features of Drug Description

We treated ADR identification as an information retrieval problem, such that our model could discover the potential relationships between each drug and the 1325 side effects recorded. We represented the prediction target of 1325 dimensions with a binary profile of elements corresponding to the presence or absence of side effects with 1 or 0, *Y* ∈ ℕ^*n*^^×1325^ with *n* being the number of drugs. Each drug was associated with 3 types of features: the chemical properties, biological properties, and information from the literature. In addition, the known ADR records of drugs were included. After preprocessing, we filtered out 2 empty properties: the Isotope Atom Count and the Undefined Bond Stereocenter Count. Subsequently, the feature of chemical properties was represented using a 15-dimensional vector, with *X*_Chem_ ∈ ℝ^*n*^^×15^ for each element. The biological properties, extracted from DrugBank, contained 4 phases of information including the carriers, enzymes (for drug metabolism), protein targets, and transporters (for drug transportation). After preprocessing, we utilized the biological information to represent each drug with the 1048-dimensional vector, which included 788 protein targets, 162 enzymes, 85 transporters, and 13 carriers, with *X*_Bio_ ∈ ℕ^*n*^^×1048^ for different action types in each element. The known ADR records of a drug played an important role in identifying potential ADRs. Thus, we leveraged this information to predict potential ADRs that appeared in 2012.

The biomedical literature played an important role in this study because it contains a large amount of information related to drugs and ADRs such as clinical notes and case reports. However, one of the issues in extracting the drug information from biomedical literature is the uncertainty regarding which words or documents represent the drug. Therefore, we trained the model to understand the semantic features of drugs from 2.3 million biomedical papers on 764 drugs introduced before 2009 by utilizing one of the most popular embedding methods Word2Vec [28-30] to model it using the skip-gram model. Subsequently, we used the vector of the drug name as the drug vector (drug2vec, D2V), *X*_D2V_ ∈ ℝ^*n*^^×400^, with *n* being the number of drugs. We observed that the features represented by D2V were more comprehensive than the intrinsic features in the experiments. However, as we used drug names as the query term, we could not identify papers related to 232 new drugs. To address drugs that were not observed during the embedding training step, we expanded the D2V by introducing a drug description mapping function. Using *V*_D2V_ to denote the word-embedding space of the drug vector existing in the training and *V*_DDV_ to denote the new drug description vector the summation of each word vector related to the new drug in the papers, the mapping function *f* (*v*): *V*_DDV_→ *V*_D2V_ was developed and parameterized by a *W*, such that *v′=Wv* for *v* ∈ *V*_DDV_ and *v′* ∈ *V*_D2V_ [31] using the least absolute shrinkage and selection operator regression for training the *W* [32]. This expansion method enabled the model to process new drugs, making it more flexible.

**Figure 1:**
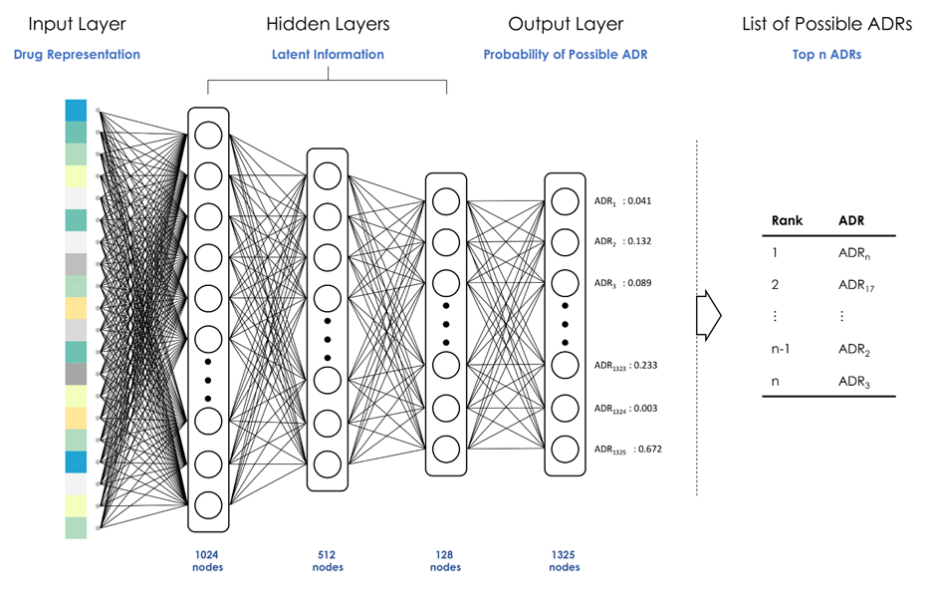
The architecture of the deep neural network model for predicting and identifying the possible adverse drug reactions (ADRs) of a drug. After predicting, we generated a list of possible ADRs of a drug by ranking the probability of ADRs from the output in the model.

**Figure 2:**
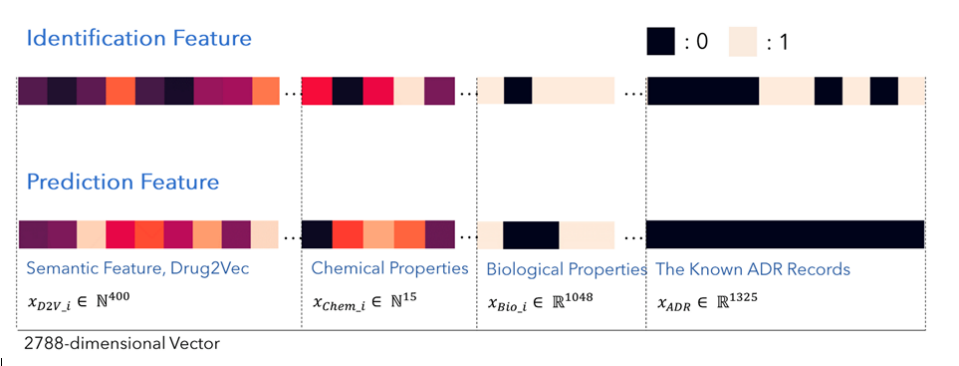
Feature representation of adverse drug reaction (ADR) identification and prediction.

### Adverse Drug Reaction Detection Deep Neural Network Model Description

We designed a DNN model that can identify and predict the ADRs of a drug with different requirements. This model (Figure 1) was based on one of the common DNN architectures, multilayer perceptron [33], which has been successfully applied in several prior studies. We added the dropout layer between each dense layer to avoid model overfitting in the training step [34,35]. The process of the nonlinear transformation in each layer ensured that the model could learn more information from the input data. The model aimed at identifying and predicting potential ADRs of a drug. The identification function is fulfilled by seeking the potential ADRs of drugs by using known records of ADRs and various features of drugs. The prediction function pertains to the detection of potential ADRs of new drugs. We designed 2 kinds of feature representations (Figure 2) to distinguish the tasks of identification and prediction. In prediction, we assumed that the ADR record for a new drug was empty. Therefore, the feature of known ADR records with zero indicated that the possible ADRs of new drugs relied only on the semantic feature (drug2vec), chemical properties, and biological properties.

We treated the identification task as an information retrieval problem because drugs may have more than one ADR. Therefore, we designed the last layer with 1325 hidden nodes, which was equal to the number of ADRs in the dataset. Evaluating the probability of ADR *y* of a given drug *x*, we defined the *p* (*y* ∣ *x*) = σ(*Wh* + *b*), σ as the sigmoid function, to transfer the hidden vector *h* to the value between 0 and 1. We learned the *p* (*y* ∣ *x*) by minimizing the cross-entropy with *D*. When θ denoted all parameters of the model, the objective function ℒ(*D*, θ) was formulated as follows:

ℒ(*D*, θ) = −*Summation* (*y_i_ log* [*p* (*y|x*)] + [*1−y_i_*] *log* [*1−p* (*y|x*]) / N

## Results

**Figure 3:**
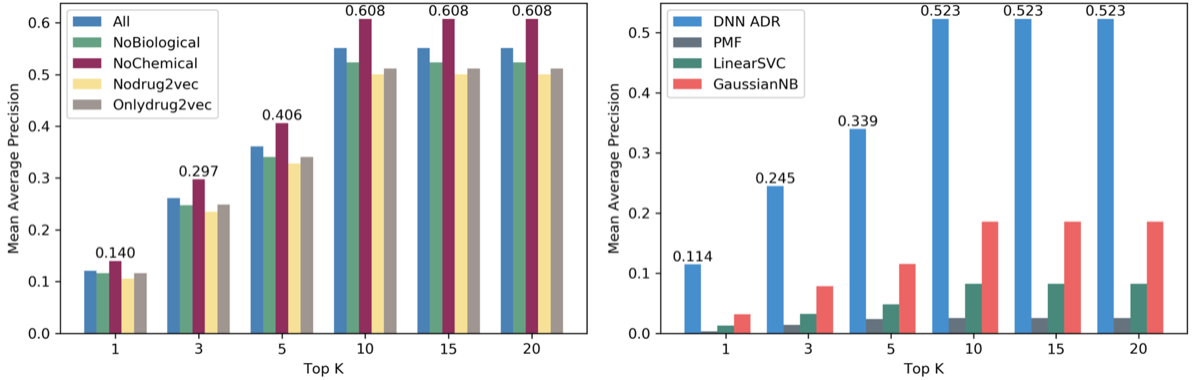
Left: Effects of different feature combinations to detect the adverse drug reactions (ADRs) of drugs; right: A comparison of our deep neural network (DNN) model with various machine learning approaches. PMF: probability matrix factorization; LinearSVC: Linear Support Vector Classifier; GaussianNB: Gaussian Naïve Bayes.

In this study, we present a detailed analysis of the performance of our DNN model. Let *Q* denote the number of drugs in the dataset. We evaluated the model using the area under the receiver operating characteristic curve, shown in Table 1, and the mean average precision (MAP) *AvePrecision* (*q*)/*Q*, which has been widely used in multilabel problem and information retrieval evaluation. First, we assessed the abilities of different feature combinations to detect the ADRs of drugs. We examined the performance of our model with reference to the features presented in Figure 3 (image on the left). The drug features included the biological, chemical, and D2V features. The chemical feature was found to perform poorly because of the duplicate and indistinguishable chemical properties extracted.

Moreover, we removed the D2V and kept the other features to train the model. The results showed that the D2V was most informative, possibly because the D2V learned the valuable information from millions of papers. We then focused on method comparison with several common methods. We compared the abilities of 3 machine learning methods, namely, probability matrix factorization (PMF), Linear Support Vector Classifier, and Gaussian Naïve Bayes [36,37], to predict and identify the ADRs of drugs. Figure 3 (image on the right) shows the performance of different models based on 5-fold cross-validation using all biological, chemical, and D2V properties as features, except PMF. The PMF exhibited the worst performance because it considered the relationship between drugs and ADRs only based on latent information. One of the reasons why our model outperforms others is that the features of a drug enrich the information via the nonlinear transformation in deep learning.

**Figure 4:**
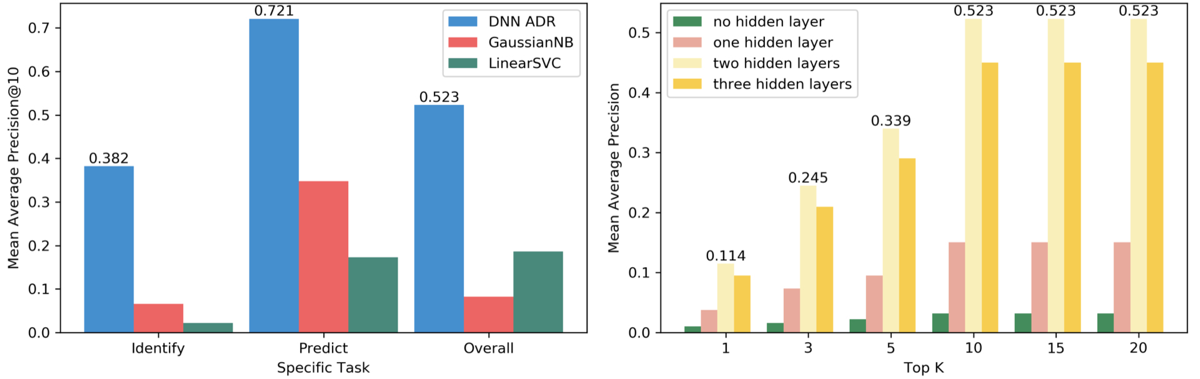
Left: Performance of the deep neural network (DNN) model on the adverse drug reaction (ADR) identification and prediction tasks and the overall performance; right: In this experiment, we showed the performance of the model with several different layers. GaussianNB: Gaussian Naïve Bayes; LinearSVC: Linear Support Vector Classifier.

Subsequently, we investigated whether our model could process the specific tasks of prediction and identification. The performance on the prediction task (Figure 4, image on the left) exceeded that on the identification task. The identification task is more difficult than prediction because the former entails the detection of potentially rare ADRs. Although the identification function of this model could be improved, its overall performance revealed its capacity to address both tasks simultaneously.

In addition, we plotted the performance of the model with a different number of hidden layers (Figure 4, image on the right). The performance of the model did not improve with an increase in the number of hidden layers. The model with 2 hidden layers was better than the others. Specifically, with the limited data size for 3 hidden layers, the model was unable to learn the good parameters from the data. Evidently, the number of hidden layers relies on data properties and the amount of data in the DNN.

To evaluate our mapping function, we examined the drug expansion through the transfer of drug description to the D2V. The results, shown in Table 2, indicated that our mapping function could fit the performance of the D2V. The performance of the drug description through the mapping function was slightly better than that of D2V, possibly because some drugs did not exist in the space of D2V. This finding indicates that this model cannot predict the ADRs of such drugs without the mapping function. Accordingly, the mapping function was found to render the model more flexible to address new drugs.

**Table 1:** The result showing the performance of model evaluated by area under the receiver operating characteristic curve (AUC).

Model	AUC
Probability matrix factorization	0.500
Linear Support Vector Classifier	0.523
Gaussian Naïve Bayes	0.597
Deep neural network adverse drug reaction (DNN ADR) without hidden layer	0.641
DNN ADR with 1 hidden layer	0.823
DNN ADR with 2 hidden layers	*0.844^a^*
DNN ADR with 3 hidden layers	0.814
DNN ADR without Bio features	0.823
DNN ADR without Chem features	0.837
DNN ADR without drug2vec features	0.803
DNN ADR	*0.844*

^a^The italicized values indicate the best results in this comparison.

**Table 2:** The results showing the ability of the mapping function to transfer the drug description to drug2vec with Mean Average Precision at Top N (MAP@N).

MAP@N	1	3	5	10	15	20
Mapping function	*0.068^a^*	*0.179*	*0.272*	*0.462*	*0.462*	*0.462*
drug2vec	0.065	0.174	0.267	0.453	0.453	0.453

^a^The italicized values indicate the best results in this comparison.

## Discussion

### Principal Findings

**Figure 5:**
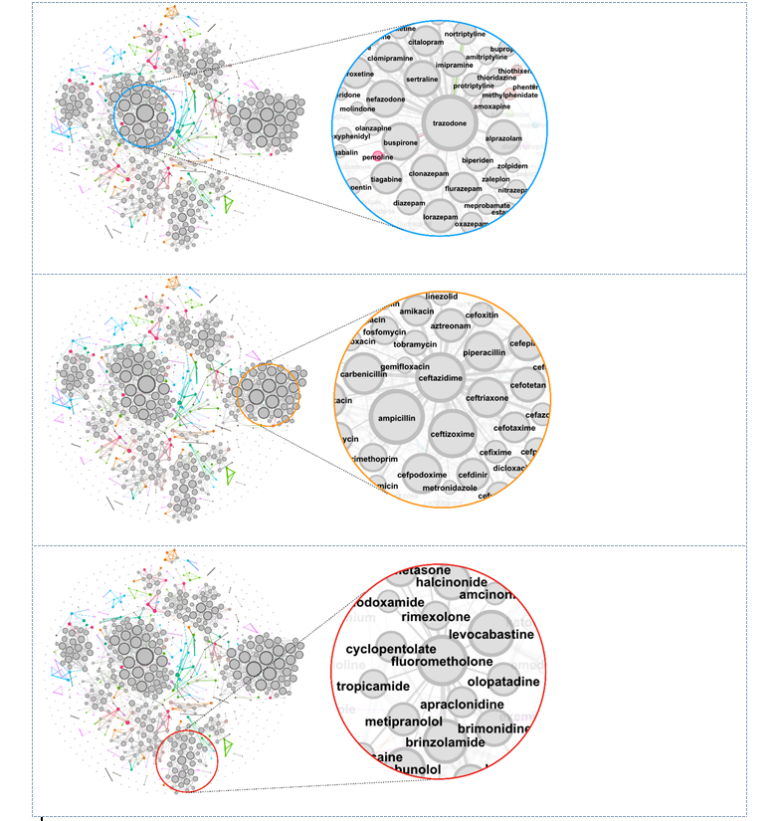
Relationship between drugs using the semantic feature (drug2vec) of the deep neural network model. There were 746 nodes in this graph, each representing a drug. The clusters indicated the drugs with a specific treatment. Top: The cluster comprised antidepressants; middle: The cluster contained antibiotics; bottom: The cluster included ophthalmic medications.

In this study, we aimed to increase the diversity of information on drugs to improve our ability to detect ADRs. Accordingly, we extracted information from the chemical and biological properties of drugs and from the existing biomedical literature. The MEDLINE was selected as the source for biomedical literature to identify important auxiliary data because it contains several types of biomedical papers, such as clinical trials, case reports, and observational studies, related to drugs. However, it was difficult to use keywords to identify specific drugs from millions of papers and words. Therefore, we utilized 2.3 million biomedical papers to identify the semantic features of drug using the skip-gram model in Word2Vec. In particular, for a central word *w*_*t*_ under consideration, the probability *p* (*w*_*t*__±__*i*_∣ *w*_*t*_) of predicting the surrounding word *w*_*t*__±__*i*_ depended on the *w*_*t*_. The subscript *t* indicates a target word, such as the drug name “Dantrolene,” and the *i* represented the windows size of the target word. This feature helped us extract the latent information of each word, including the words comprising the drug’s name. After this word-embedding training, we investigated whether the semantic feature (D2V) could represent the properties of drugs, such as biomedical and chemical properties. We visualized the relationships between the learned drug-embedding vectors based on their similarity (Figure 5). This graph included 746 drugs and presented the relationship of these drugs with those of the latent information learned from the semantic feature. Each node presents a drug and the edge represents the similarity between other drugs. The more similar the drugs in each pair were, the closer they were in the graph. The larger nodes represented the drugs that were more similar to other drugs. We found that the model seems to cluster the drugs used in specific treatments.

For instance, the drugs in the cluster with the blue circle shown in Figure 5 (image on the top) comprised antidepressants such as trazodone, citalopram, clomipramine, and paroxetine. The cluster to the right contained antibiotics such as ampicillin, ceftazidime, cefpodoxime, and cefotaxime shown is Figure 5 (image in the middle). Moreover, the cluster at the bottom included ophthalmic medications such as fluorometholone, levocabastine, brinzolamide, and dipiverfrin (Figure 5, image at the bottom). There were other small clusters with their own specific treatment. Thus, we learned the relationships among drugs and the latent information from the papers included in the embedding function at the text-level. Accordingly, the semantic feature had a great effect on the performance of our model.

Subsequently, we examined the ability of this model to perform its identification and prediction functions with reference to serious ADRs defined by the Micromedex. Using the identification function of the model, we ranked the potential ADRs in a list by the probability of their occurrence (Table 3). One of the reasons why the probability of ADR presented in Table 3 was not highly prominent was that the positive samples of these ADRs were rarely reported in this dataset. However, our model could identify that hydroxychloroquine led to muscle cramps, which is a serious ADR that occurs in severe neuromuscular disease. In addition, we extracted 5 drugs that were recorded only in 2012 to examine the prediction function of this model.

**Table 3:** The adverse drug reaction (ADR) prediction and identification results of the model.

Drug		Serious ADR	Rank	Probability
**Identification results of drugs with known ADR records**				
	Dantrolene	Anemia	12	0.012
	Dantrolene	Congestive heart failure	15	0.009
	Hydroxychloroquine	Muscle Cramp	1	0.997
	Hydroxychloroquine	Photophobia	16	0.017
	19-nortestosterone	Serum cholesterol raised	4	0.150
	Carbachol	Retinal detachment	3	0.690
**Prediction results of drugs without ADR records**				
	Atazanavir	Anemia	17	0.920
	Carbinoxamine maleate	Agranulocytosis	14	0.453
	Carbinoxamine maleate	Anemia, Hemolytic	16	0.340
	Darunavir	Hyperglycemia	20	0.750
	Temsirolimus	Infection	20	0.974
	Zoladex	Myocardial infarction	7	0.961
	Zoladex	Hypersensitivity	12	0.920

Findings revealed that our model has the capacity to predict the serious ADRs of new drugs. For instance, the model predicted that Zoladex could lead to a serious ADR, myocardial infarction, which is one of the commonest causes of death in developing countries.

### Limitations

This study has several limitations that need to be addressed in future studies. First, data diversity plays an important role in the model. We only used the data published in SIDER. Our model will be more persuasive and reliable if we can include more data from different datasets. Because the chemical and biological properties of drugs contribute most to their effects on human, the more the databases of drug properties included, the better the performance of our model. On the other hand, if we have access to more open-source data, including clinical trials, spontaneous reporting systems, and EMRs with support from government and pharmaceutical industry, our model will have better prediction. Furthermore, our model focused on the ADR prediction and identification. To identify the probability of occurrence of each ADR, we set 1325 hidden nodes and the total number of ADRs in the dataset in the output layer. In other words, although we had a mapping function to address new drugs, this model could only predict existing ADRs. Therefore, in the future work, we plan to utilize more detailed features such as drug-ADR interaction [13], drug-drug interaction, and ADR-ADR interaction networks for the prediction of ADRs. Furthermore, we also plan to investigate other embedding approaches to represent the ADRs to help predict the relationships between drugs and new ADRs.

### Conclusions

We developed a novel ADR detection model based on the biological and chemical properties of drugs and the D2V (the semantic feature). After discussing the drug similarities with domain experts from the National Cheng Kung University Hospital and the Institute of Clinical Pharmacy and Pharmaceutical Sciences, we found out that the D2V can represent a characteristic of the drug. Our model could not only discover the potential ADRs of drugs but also predict the possible ADRs of new drugs. To discover potential ADRs based on the previous records, our model could identify the hidden relationship between ADR-ADR interactions. Furthermore, to predict the possible ADRs of a new drug without any previous ADR records, using the D2V feature, our mapping function exhibited good profiling for transferring the drug description into the D2V. The model exhibited good performance on both tasks and generated the most suitable results. It will help pharmacists and health care providers to understand the potential risk of side effect of drugs and address the issue of underreporting of spontaneous reports. Above all, our model will aid pharmacovigilance by identifying and predicting potential ADRs.

## Abbreviations

ADE: adverse drug events
ADR: adverse drug reaction
AUC: area under the receiver operating characteristic curve
DNN: deep neural network
EMR: electronic medical records
MAP: mean average precision
MEDLINE: Medical Literature Analysis and Retrieval System Online
PMF: probability matrix factorization
SIDER: Side Effect Resource
